# Simultaneous sealing and bisection of porcine renal blood vessels, ex vivo, using a continuous-wave, infrared diode laser at 1470 nm

**DOI:** 10.1007/s10103-024-04093-0

**Published:** 2024-06-22

**Authors:** Woheeb M. Saeed, Jude K. Yoshino, Alexandria J. Traynham, Nathaniel M. Fried

**Affiliations:** 1 Department of Physics and Optical Science, University of North Carolina at Charlotte, 9201 University City Boulevard, Charlotte, NC 28223 United States; 2 Department of Mechanical Engineering, University of North Carolina at Charlotte, Charlotte, United States

**Keywords:** Bisection, Blood vessels, Infrared laser, Sealing

## Abstract

Electrosurgical and ultrasonic devices are used in surgical procedures for hemostatic sealing and bisection of vascular tissues. Previous benchtop studies alternatively demonstrated successful infrared laser sealing and cutting of blood vessels, in a sequential, two-step approach. This study describes a smaller, laparoscopic device compatible design, and simultaneous approach to sealing and bisection of vessels, with potential optical feedback. A 1470-nm infrared diode laser sealed and bisected 40 porcine renal arteries, ex vivo. A reciprocating, side-firing, optical fiber, housed in a transparent square quartz optical chamber (2.7 × 2.7 × 25 mm outer dimensions), delivered laser energy over an 11 mm scan length, with a range of incident powers (41–59 W) and treatment times (5–21 s). Vessel diameters ranged from 2.5 to 4.8 mm. Vessel burst pressure measurements were performed on each cut end (*n* = 80) with success indicated by pressures exceeding 360 mmHg. All vessel ends were successfully sealed and bisected (80/80). The highest incident power, 59 W, yielded short treatment times of 5–6 s. Peak temperatures on the external chamber surface reached 103 ^o^C. Time to cool down to body temperature measured 37 s. Infrared lasers simultaneously seal and bisect blood vessels, with treatment times comparable to, and temperatures and cooling times lower than reported for conventional devices. Future work will focus on integrating the fiber and chamber into a standard 5-mm-outer-diameter laparoscopic device. Customization of fiber scan length to match vessel size may also reduce laser energy deposition, enabling lower peak temperatures, treatment times, and cooling times.

## Introduction

Energy-based devices are used in about 80% of the 15 million laparoscopic surgical procedures performed globally each year [1, 2]. Ultrasonic (US) and radiofrequency (RF) devices are commonly used to provide hemostatic sealing and bisection of vascular tissues in a variety of laparoscopic procedures. Experimental infrared (IR) laser sealing and/or bisection of vascular tissues has recently been reported using a high power, 1470-nm, diode laser [3–11]. Potential advantages based on these previous pre-clinical IR laser studies include: (1) rapid optical sealing and cutting of vascular tissues without use of a separate deployable mechanical blade to bisect tissue seals, (2) less thermal spread for potential use near sensitive tissues (e.g. nerves), (3) higher vessel seal strengths, and (4) lower device jaw temperatures for providing a safer profile (e.g. to avoid thermal damage to adjacent soft tissues from device contact) and to enable shorter device cooling times in between applications for reduced operating room times and cost savings.

A major design constraint is the 5-mm-outer-diameter (OD) shaft and distal jaws (end effectors) of conventional laparoscopic, energy-based, surgical devices. The bottom, stationary or fixed jaw design is required to reflect the IR laser beam at a 90^o^ angle, and also convert the circular spatial beam profile into a linear beam pattern, to create a uniform lengthwise seal across the width of the blood vessel, all within this limited space. In general, the top, active, or grasping jaw, utilizing a pivoting hinge, opens and closes for grasping and compressing vessels to facilitate thermal sealing. Previous laboratory studies characterized a reciprocating, side-firing, optical fiber to produce a uniform linear beam profile for thermal vessel seals within a 5-mm-OD design [9].

Another design limitation is the metallic materials used for jaws in standard laparoscopic devices. The limited surgical field-of-view provided by these non-transparent materials may make accurate positioning and centering of tissues within the jaws more difficult to achieve in clinical practice. Recent laboratory studies directly compared quartz and sapphire transparent optical chambers, for future integration into a laparoscopic device [11]. Use of an optically transparent chamber may enable improved visibility for positioning vascular tissues within the device jaws. This approach may also enable customization of the reciprocating fiber scan length to match the compressed width of the blood vessels. In preliminary studies, quartz was determined to be a superior material to sapphire, based on more consistent vessel seal strengths, shorter device cooling times, as well as cost and availability [11].

Previous experimental studies also demonstrated rapid and precise sealing and bisection of blood vessels, ex vivo, in a two-step, sequential approach [4]. However, the large benchtop system used standard bulk optics, which are not practical for future laparoscopic surgery in animal studies, in vivo. This study utilizes a compact, square quartz optical chamber (2.7 × 2.7 × 25 mm OD) determined to be preferable during previous vessel sealing studies [11].

The main objectives of this study are two-fold, (1) to investigate the feasibility of simultaneous IR laser vessel sealing and bisection in a one-step approach, using a quartz optical chamber suitable for integration into a laparoscopic device, and (2) to test the feasibility of using the optical signal originating from the therapeutic laser and transmitted through the cut vessel, as a closed-loop, optical feedback system for immediately deactivating the IR laser upon successful vessel bisection.

## Methods

### Tissue preparation

Fresh porcine kidney pairs were acquired from an abattoir (Animal Technologies, Tyler, TX). Renal arteries were dissected and stored in physiological saline in a refrigerator prior to use. A total of *n* = 10 blood vessels, each with a similar uncompressed mean diameter of 3.2 ± 0.5 mm, 3.4 ± 0.6 mm, 3.4 ± 0.8 mm, and 3.3 ± 0.7 mm, were selected for each group of four laser power settings (41, 47, 53, and 59 W), respectively (*P* = 0.84).

### Laser parameters

**Fig. 1:**
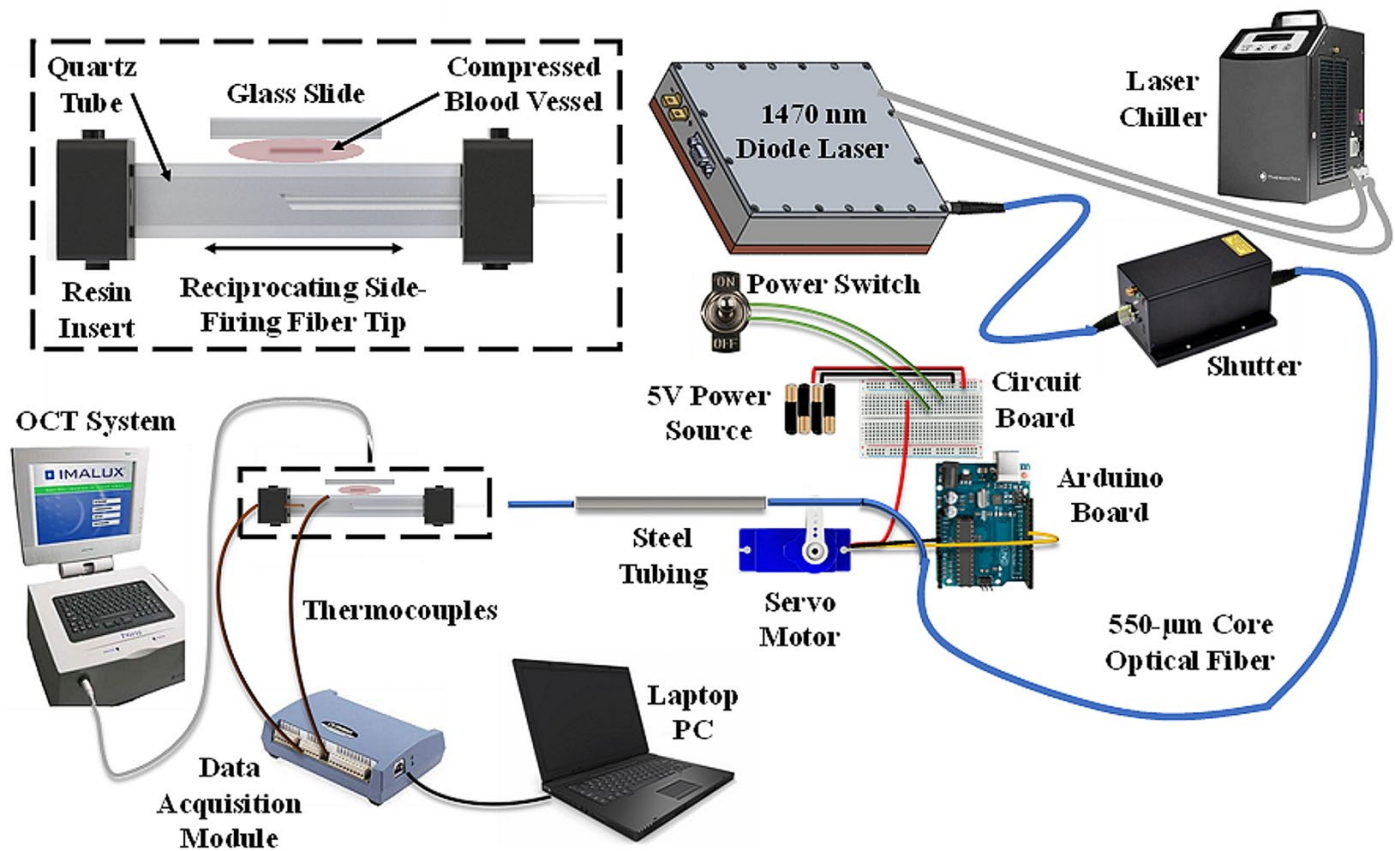
The laser was connected to a side-firing fiber. Two mounts (not shown) prevented the fiber from rotating. An Arduino board was pre-programmed to a specific scan length and speed for the servo motor. The fiber was threaded through and locked down onto an arm attached to the servo motor. The servo motor was battery powered, with an external ON/OFF switch. The lower jaw consisted of steel tubing attached to quartz square tubing (25-mm-long with 2.7 × 2.7 mm OD and 1.8 × 1.8 mm ID). Black resin plugs sealed the optical chamber on proximal and distal ends, with small holes on each end for insertion of the bare optical fiber (with jacket removed), and insertion of the micro-thermocouple, respectively. The black plug on the distal end additionally served to absorb stray light in the forward direction. Vessel samples were compressed onto the quartz tubing using a glass slide, simulating a transparent upper jaw

A 100-Watt, 1470-nm wavelength, IR diode laser (BrightLase, QPC Lasers, Sylmar, CA) was used for the vessel sealing studies. The laser was operated in continuous-wave (CW) mode with incident power of 41, 47, 53, and 59 W and irradiation time ranges of 20–21, 15–17, 10–11, and 5–6 s, respectively. Laser power output was calibrated using a power meter (EPM1000, Coherent, Santa Clara, CA) and detector (PM-150, Coherent). A high-power fiber optic shutter (SH-200-55-1470-M-O-T-BH-SP, Oz Optics, Ottawa, Canada) was connected by a fiber optic patch-cable on one end to the laser diode module, and the other end to a surgical fiber (Fig. 1). The shutter enabled ramp up of the laser power output prior to initiating the procedure as well as stable power output during laser irradiation. Losses at multiple optical component interfaces (e.g. fiber/air interfaces) and coupling through the fiber optic shutter, limited maximum power output through the surgical fiber to 59 W.

Blood vessel samples were compressed and fixed in place using a 0.5-mm-thick optical window locked in a clamp, to simulate a transparent surface for the upper jaw (Fig. 1). An optical coherence tomography (OCT) system (Niris, Imalux, Cleveland, OH) with 2.7-mm-OD probe provided non-invasive measurement of the compressed vessel thickness to confirm consistent pressures and reproducible measurements between samples. Compressed tissue thickness was fixed at 0.4 mm to closely match the optical penetration depth of IR light in water-rich soft tissues at 1470 nm, and to provide uniform, full-thickness seals. The OCT probe was placed next to the sample and optical window, to non-invasively image the tissue thickness, and then moved away before laser activation. The OCT system operated at 1310 nm, with axial and lateral resolutions of 11 and 25 μm. Images had 1.6 mm and 2.0 mm axial and lateral dimensions. The combined thickness of the compressed tissue (0.4 mm) plus optical window (0.5 mm) was less than the OCT axial scan depth of 1.6 mm, enabling measurement of compressed vessel thickness.

### Side-firing fiber preparation

A low-OH, silica optical fiber (FG550LEC-CUSTOM, Thorlabs, Newton, NJ) with 550-µm-core, 600-µm-cladding, 1040-µm-jacket, and numerical aperture of 0.22 was used. The proximal fiber tip with high-power SMA905 connector was attached to the shutter. The side-firing, angled distal fiber tip was prepared using a fiber optic polisher (Radian™, Krelltech, Neptune City, NJ), to achieve a 50^o^ polish angle and 90^o^ light delivery.

### Fixed optical chamber assembly

The optical chamber consisted of quartz (Technical Glass Products, Painesville Township, OH) square tubing with dimensions of 1.8 × 1.8 mm ID, 2.7 × 2.7 mm OD, 25 mm length, and 0.45 mm wall thickness. A 3D-printed, black resin plug (RS-F2-GPBK-04, Formlabs, Durham, NC) was placed on each end of the chamber (Fig. 1). The proximal plug had a small hole to allow insertion of the fiber. The distal plug also had a small hole to allow insertion of a thermocouple (TC), but otherwise provided fluid-tight closure and absorbed stray light in the forward direction. The fiber was inserted into the quartz tubing and clamped in place, leaving a 0.6 mm space between the fiber tip and the inner walls of the tubing. The distance from fiber tip to vessel wall measured 1.05 mm (air gap of 0.6 mm + quartz wall thickness of 0.45 mm).

### Optical beam characterization

The side-firing fiber output beam was directed towards a detector (PM100-19 C, Coherent) connected to a power meter (EPM2000, Coherent). An XYZ stage (460 A-XYZ, Newport, Irvine, CA) with mounted razor blade was used. To accurately simulate the distance from fiber tip to tissue sample, the razor blade was set in front of the fiber, 1 mm from its edge. The razor blade was moved across the laser beam in 100 μm increments, and power recorded at each location, for beam characterization.

### Reciprocating fiber setup

Figure 1 shows the reciprocating, side-firing, fiber experimental setup. A high-power connector on the proximal fiber end was attached to the shutter. Two custom mounts (not shown) and steel tubing prevented the fiber from rotating and becoming misaligned. A micro-controller (Uno, Arduino, Boston, MA) was pre-programmed to a specific scan length (11 mm) and speed (87 mm/s) for the servo motor (SG90, Deegoo-FPV, China). The microcontroller had a custom uploaded code that allowed the stepper motor to sweep back and forth over an angle of 45^o^ with a 2.5 ms delay between steps. The motor used 4.8 V, giving 1.8 kg-cm in stall torque at 0.10 s per 60^o^. The fiber was threaded through and locked down onto an arm attached to the motor. The motor was powered by a battery pack, with a circuit board enabling an external on/off switch. Blood vessels were compressed onto the quartz chamber using a glass microscope slide as an optical window, simulating a transparent surface for the upper jaw.

### Temperature measurements

Two micro-thermocouples (5TC-TT-T-36-72, Omega, Norwalk, CT) with 125-µm-OD were used: one TC was placed inside the quartz and another TC placed in contact with the chamber’s external surface on the opposite side to the exiting laser beam. The TC tip extended 1 mm beyond the plug, within the tubing, but beyond the laser beam path. A personal computer with temperature software (TracerDAQ + InstaCal, Omega) recorded TC temperatures as a function of time (Fig. 1).

### Burst pressure (BP) measurements

The vessel burst pressure setup for measuring vessel seal strengths consisted of a pressure meter (717 100 G, Fluke, Everett, WA), infusion pump (78–01000 C, Cole Parmer, Vernon Hills, IL), and iris clamp (ID25, Thorlabs) [3–11]. The vessel lumen was clamped over a cannula attached to the pump. Deionized water was flowed at 100 ml/hr and maximum BP recorded. A successful seal exceeded 360 mmHg, or three times systolic blood pressure (120 mmHg), consistent with industry standards for testing.

### Optical transmission measurements

A similar 550-µm-core optical fiber as for the IR laser sealing/cutting experiments, was also used for optical transmission measurements, but the bare distal fiber tip was instead polished flat. A total of 8 blood vessels (d = 3.1 ± 0.6 mm) were tested. The IR laser was operated in CW mode at an incident power of 6.5 W, producing a circular beam diameter of 0.560 mm, and yielding an irradiance of 26.4 W/mm^2^ or 2640 W/cm^2^, at the vessel surface. The working distance between the fiber tip and tissue surface was kept fixed at 1.3 mm. The tissue was compressed between two acrylic windows to a 0.4 mm thickness. A 1.5-mm-diameter hole was drilled in the center of each acrylic window, to enable the optical signal to be transmitted directly through the tissue to the detector, without additional Fresnel reflection losses.

**Fig. 2:**
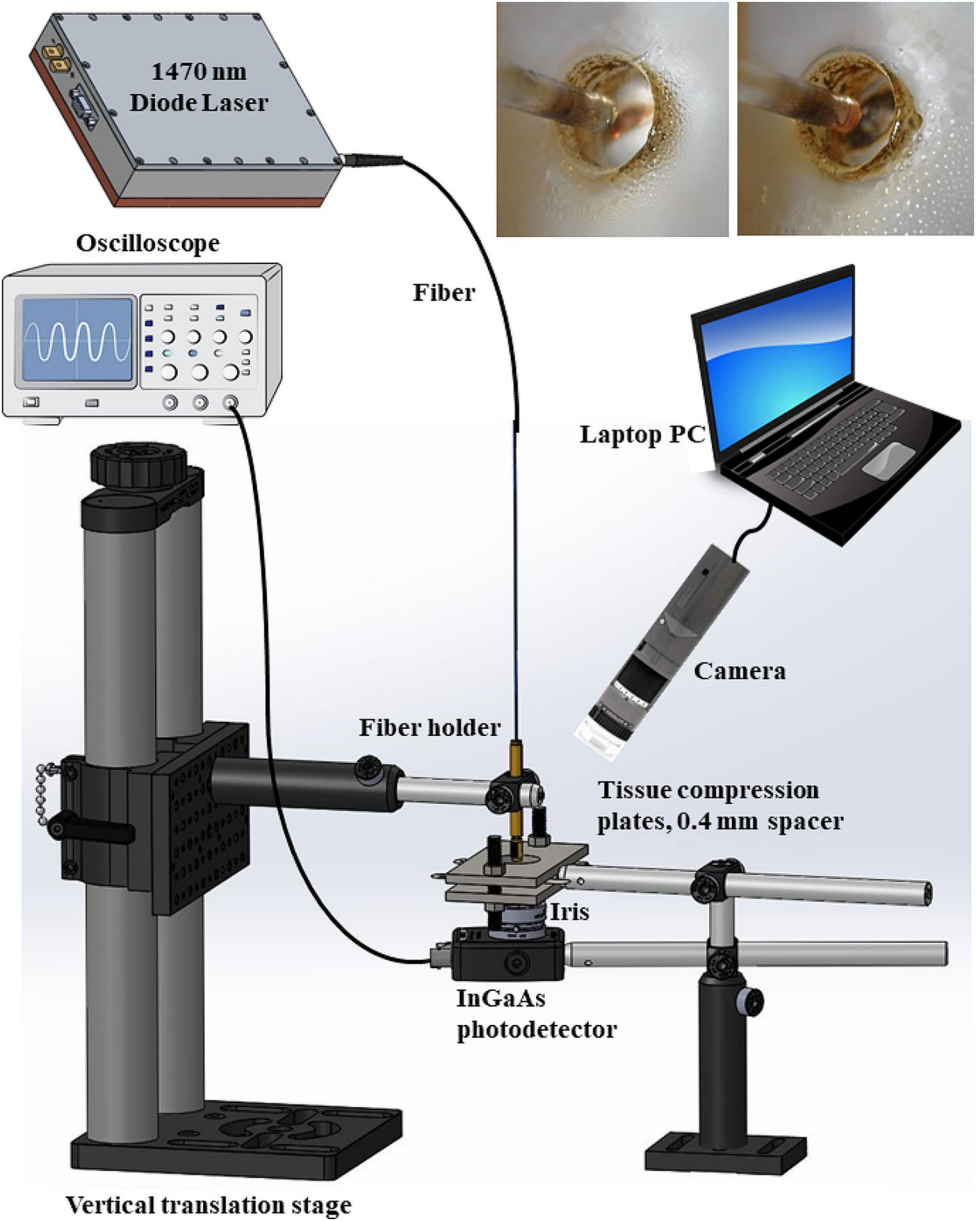
Experimental setup for acquiring the optical transmission signal during continuous-wave laser ablation of compressed porcine renal blood vessels. (Left top inset) Start of carbon layer formation on the tissue surface during laser irradiation. (Right top inset) Formation of an ablation crater during laser irradiation

Figure 2 shows the experimental setup. The optical transmission signal was acquired by an InGaAs photodiode detector (PDA400, Thorlabs) connected to an oscilloscope (TDS 2002b, Tektronix, Beaverton, OR). A neutral density filter (not shown) with an optical density of 4.0 (NE40, Thorlabs) was placed between the tissue and photodetector to attenuate the signal and protect the photodetector. An iris (SM1D12C, Thorlabs) with 1.5-mm-diameter opening was also placed in front of the photodetector to reduce contributions from multiple scattered light to the transmission signal. The laser was de-activated once the oscilloscope signal saturated, corresponding to an ablated hole in the tissue. A compact camera (AF4915ZT, Dino-lite, Torrance, CA) recorded video of each experiment.

## Results

### Optical characterization of side-firing Fiber

Side-firing optical fibers polished at a 50^o^ angle delivered 94% of light sideways at a 90^o^ angle, with 2.3% in the forward (0^o^) direction and 3.7% in the − 90^o^ direction. Total Fresnel reflection losses for the two quartz-air interfaces measured 6.6%, with 93.4% of the side-firing light transmitted through the quartz tubing wall [11]. A razor blade scan was performed at a distance of 1 mm from the fiber, corresponding to where the blood vessel sample would be located on the external surface of the quartz tubing. The 1/e^2^ beam spot size measured 600 × 800 μm.

Using the elliptical beam dimensions of 0.06 × 0.08 cm at the tissue surface, scanned over a length of 1.1 cm, it is possible to estimate the total fluence (J/cm^2^) delivered to the tissue by the reciprocating beam, for each incident power and irradiation time. An average uncompressed vessel diameter of about 0.33 cm used in these studies, and a typical 50% increase in vessel width with compression previously reported by several research groups [12, 13] to 0.50 cm, yields a correction factor of (0.50 / 1.1), or 0.45. Thus, the laser beam scan length of 1.1 cm is only incident on the compressed tissue sample width of 0.5 cm, 45% of the time. The rectangular beam area for the entire scan length is given by the measured beam width (0.08 cm) times laser scan length (1.1 cm), or 0.088 cm^2^. Hence, total fluence (F) delivered to the vessel for each laser power group and fluence level is calculated to be:

F = [(0.45) (41 W) (20.5 s)] / 0.088 cm^2^ = 4,298 J/cm^2^.

F = [(0.45) (47 W) (16 s)] / 0.088 cm^2^ = 3,845 J/cm^2^.

F = [(0.45) (53 W) (10.5 s)] / 0.088 cm^2^ = 2,846 J/cm^2^.

F = [(0.45) (59 W) (5.5 s)] / 0.088 cm^2^ = 1,659 J/cm^2^.

This large difference in total fluence can be explained by the continual loss of heat through thermal conduction during the longer laser irradiation durations, thus requiring greater total fluence to achieve the threshold temperatures needed for tissue ablation. Note that thermal conduction of heat during laser irradiation is also desirable as it contributes to thermal sealing of the vessel.

### Burst pressure (BP) measurements

Vessel burst pressures (mmHg) were conducted for all vessels sealed and bisected (*n* = 40) at four different laser incident power levels and irradiation times. All vessel cut ends tested (80/80) withstood BPs above three times systolic pressure (360 mmHg), yielding a 100% success rate. The shortest irradiation time was 5–6 s at 59 W. The BP data is summarized in Table 1.

**Table 1:** Burst pressures (BP) of both bisected segments (S1/S2) of blood vessels for each incident power and irradiation time

Incident Laser Power (W)	Irradiation Time (s)	Mean BP (mmHg)
41	20–21	S1: 788 ± 374 / S2: 758 ± 320
47	15–17	S1: 1046 ± 355 / S2: 1053 ± 320
53	10–11	S1: 1199 ± 149 / S2: 875 ± 421
59	5–6	S1: 984 ± 351 / S2: 976 ± 350

**Fig. 3:**
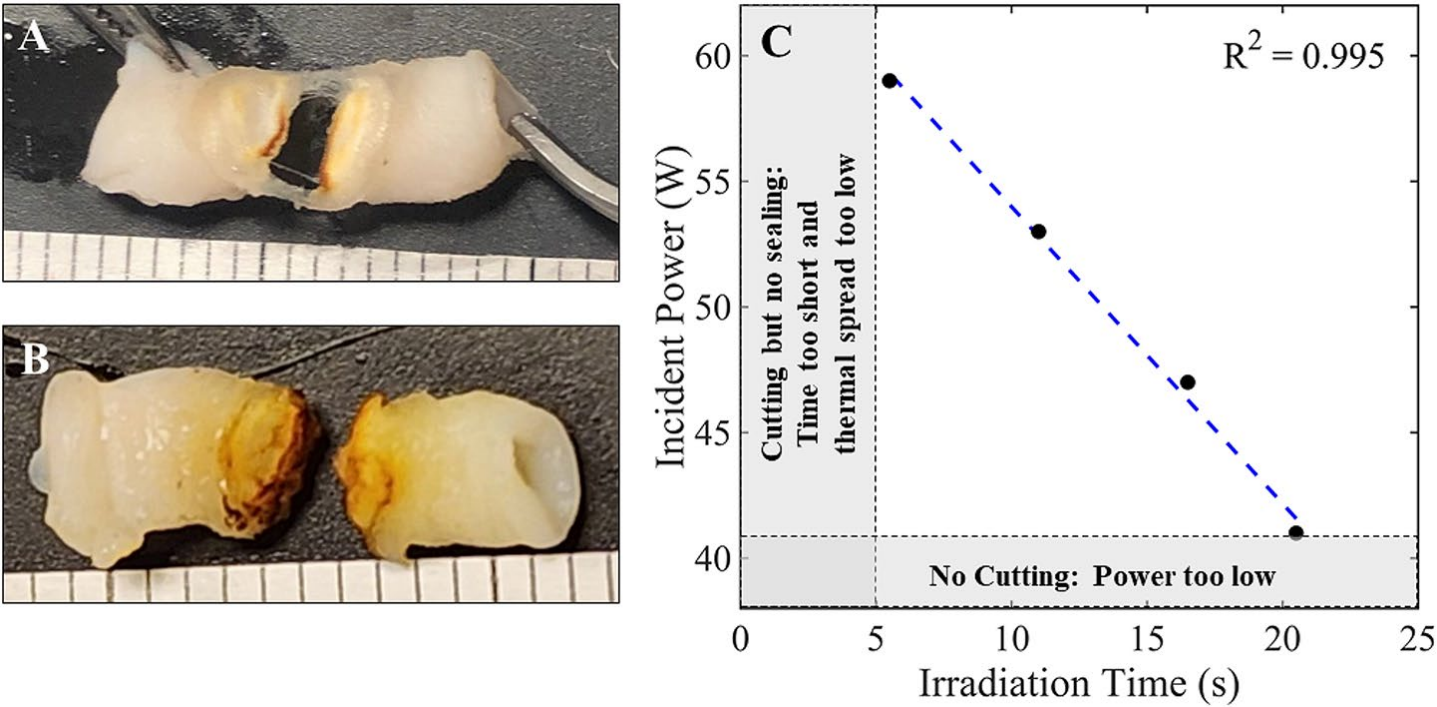
(**A**) Photograph of a vessel after bisection using 59 W for 5 s. A fascia layer is visible and the vessel was easily detached by pulling uniformly from both sides. (d = 3.2 mm, BP1: 460 mmHg, BP2: 776 mmHg). (**B**) Photograph of a vessel after bisection using 59 W for 6 s. A clean cut is shown (d = 3.5 mm, BP1: 1160 mmHg, BP2: 372 mmHg). (**C**) Comparison of laser incident power and irradiation times for each data set. Laser irradiation times shorter than 5 s resulted in tissue cutting, but not sealing, presumably due to insufficient thermal conduction and thermal spread during the laser treatment time. Laser incident powers less than 41 W failed to produce a full-thickness cut, due to insufficient irradiance

Representative images of a blood vessel sample before and after bisection are shown in Fig. 3AB. Laser irradiation times shorter than 5 s resulted in tissue cutting, but not sealing, presumably due to insufficient thermal conduction and thermal spread during the laser treatment time. Laser incident powers less than 41 W failed to produce a full-thickness cut, due to insufficient irradiance. Otherwise, there was a strong linear fit to the power/time data points, demonstrating that higher laser incident power enables shorter laser irradiation times, as expected (Fig. 3C).

### Thermal characterization of quartz jaw

Temperature-time data for micro-thermocouples placed on the external (T_out_) and internal (T_in_) surfaces of the quartz chamber was also collected using the shortest irradiation time of 5–6 s and incident power of 59 W. The maximum temperature measured on the internal chamber surface, 77 ^o^C, was lower than on the external surface, 103 ^o^C. Cooling times for the external chamber surface are of much interest, since they determine how long the surgeon must wait in between successive activations of the laparoscopic device. The external surface of the quartz chamber cooled to body temperature (37 ^o^C) in 37 s.

### Optical transmission signal during blood vessel ablation

**Fig. 4:**
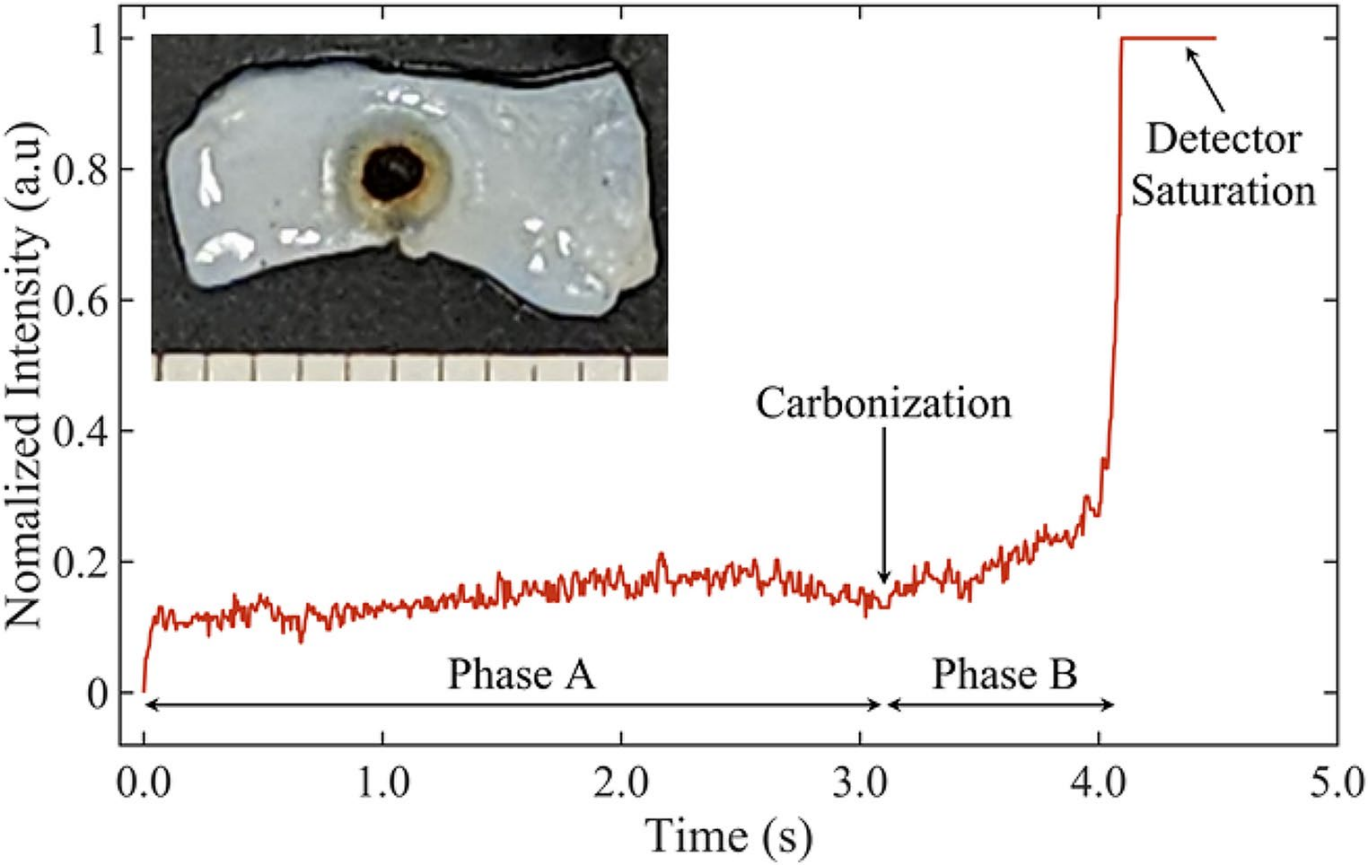
Optical transmission signal as a function of time during CW laser tissue ablation and (inset) the representative tissue sample (d = 3.2 mm)

Optical transmission measurements revealed two distinct phases (Fig. 4). Phase A is the period between the start of CW laser irradiation, with corresponding dehydration and coagulation of the tissue, and the beginning of carbon formation on the vessel surface. Analysis of the videos showed that the tissue surface discolored due to shrinkage from water evaporation and thermal denaturation. During phase B, tissue carbonization begins to form and grow in size. A rapid increase in optical transmission and eventual saturation of the signal occurs, as the tissue is vaporized and a full-thickness ablation crater forms. The laser was de-activated when the signal saturated. This steep and rapid rise in signal at the photodetector, corresponding to tissue perforation, may serve as a potential optical feedback system during laser bisection of vessels. Appendix A also provides calculations for the ablation velocity, based on these experiments.

## Discussion

### Vessel sealing and bisection studies

Previous benchtop studies were performed on a large scale, with bulk optical components unsuitable for integration into standard laparoscopic devices. These previous studies also utilized a two-step process with sequential sealing and then cutting of the vessel, involving a more complex method of translation of the optical components to change the beam focus and laser spot size [4]. This study investigated the feasibility of simultaneous IR laser vessel sealing and bisection of porcine renal arteries in a one-step approach, using a compact quartz optical chamber suitable for integration into the distal end effector jaws of standard 5-mm-OD laparoscopic devices.

It should be noted that previous computer simulations of the laser irradiation distribution through the quartz chamber [11], showing a Gaussian-like beam profile, with additional steps on the wings, motivated this approach to simultaneous sealing and bisection of vessels, based on the ability to coagulate and seal at the periphery of the beam and ablate or cut in the center of the beam. Previous laboratory studies also demonstrated successful sealing of blood vessels at 30 W for 5 s at 1470 nm [11]. These parameters were therefore chosen as a baseline, and the laser power was elevated in steps with different irradiation times, and BPs were measured to confirm success.

Table 1 shows the shortest irradiation times required at each power level to simultaneously seal and bisect the vessel. It should be noted that the irradiation times contain a small range rather than a singular value due to several factors, including variable amount of fat and water content in vessels, different amounts of collagen/elastin levels (C/E ratio) and variable vessel sizes. Larger blood vessels had thicker walls which made it more difficult for the chamber to create equal pressure across the vessel and to efficiently compress it to the desired 0.4 mm thickness to match the optical penetration depth of the 1470-nm laser energy in tissue. These factors also explain in part the large range of BPs measured in Table 1.

It should be emphasized that several previous studies have also reported wide ranges in BP values using RF, US, and IR sources [6, 14–16]. In this study, the lowest laser irradiation time in each range provided a complete cut, but with a small amount of fascia remaining, easily manually pulled apart with tweezers in the laboratory or the device jaws in a clinical situation (Fig. 3A). The highest irradiation time within each range provided a complete cut with no remaining fascia strands (Fig. 3B).

The highest incident power, 59 W, yielded treatment times of 5–6 s, comparable to conventional RF and US devices. For example, a recent study reported energy activation durations of 7.7 s and 7.9 s for US and RF devices, respectively [17].

Peak temperatures and cooling times for the external surface of the chamber are also important, since they determine the safety profile of the device as well as the time the surgeon must wait between successive activations of the laparoscopic device. Peak temperatures measured 103 ^o^C on the outside of the quartz chamber at 59 W and 5–6 s. Furthermore, the external surface of the chamber cooled to body temperature (37 ^o^C) in 37 s. These values also appear promising, when directly compared with values reported for conventional RF and US devices. For example, a recent study reported that median external jaw temperatures were 126 ^o^C for RF devices and 218 ^o^C for US devices [17]. The quartz chamber’s cooling time of 37 s also compares favorably with reported mean cooling times of 54 s and 68 s for two different RF devices [18].

### Optical signal transmission studies

This study also demonstrated the feasibility of using the optical transmittance signal during IR laser tissue ablation as a closed-loop, optical feedback system for immediately deactivating the laser upon successful bisection. Optical transmission analysis during the ablation study revealed slightly variable pre-ablation times (Phase A), due to differences in tissue size, fat content, water content and C/E levels between samples. Consequently, an optical feedback system that deactivates the laser upon ablation is desired. Phase A in Fig. 4 includes the phenomena of water absorbing laser light, followed by its evaporation and tissue coagulation, which results in the decrease of signal right before the start of phase B. This decrease is believed to be associated with a rise in the scattering coefficient of the tissue surface due to thermal coagulation, which is a commonly observed property [19].

Phase B starts with carbonization and ends when the tissue ablation is complete. The start of tissue carbonization was observed as a thin layer of blackened tissue at the surface (Fig. 2 inset). Carbonization enhances light absorption, leading to a reduction in light transmission. Nevertheless, the creation of carbonized tissue did not lead to a reduction in transmission in the forward direction. Video frames displayed a progression where initially, a limited central area of carbonized tissue was generated. This area then transformed into a growing ring, producing an ablation hole in the tissue center.

Based on these observations, we hypothesize that upon carbonization, the tissue rapidly vaporized, forming a small ablation hole because of significantly increased absorption. As the carbonized zone expanded outward in the form of a growing ring, the hole diameter increased (Fig. 2). This sequence of events could potentially explain the absence of any observed decline in light transmission, as reported in previous studies [20]. Consequently, when the tissue is completely vaporized and the hole is complete, the signal saturates, and the laser can be de-activated (Fig. 4).

### Prototype device design

**Fig. 5:**
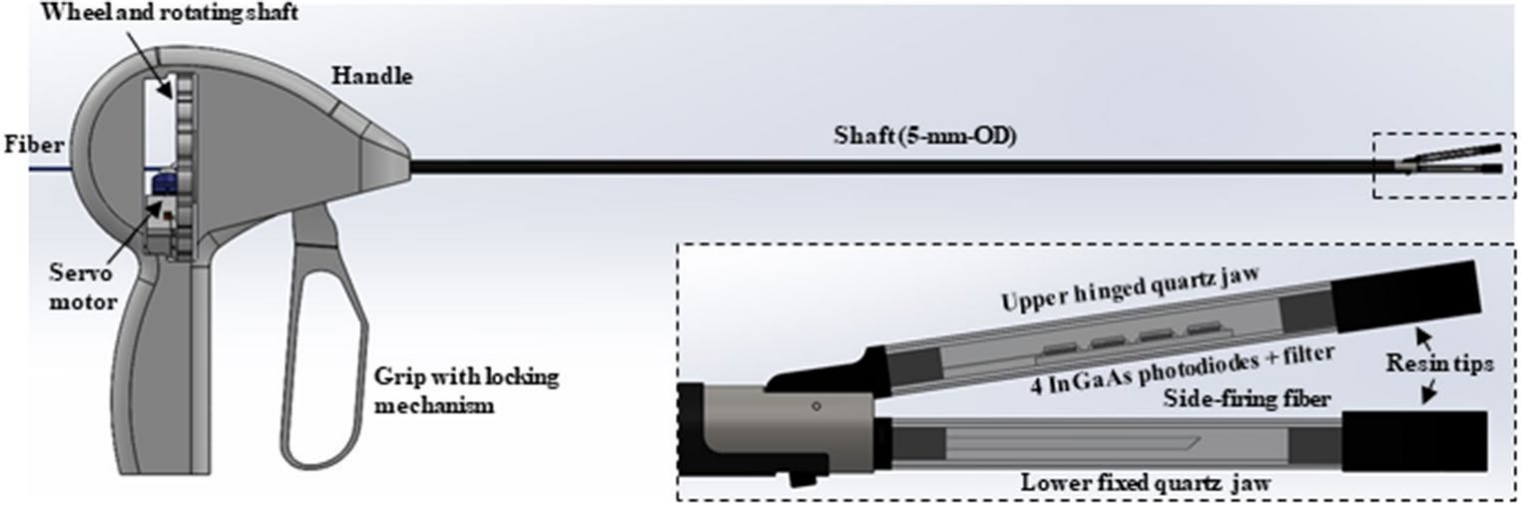
Diagram of proposed laparoscopic device with quartz jaws and optical feedback system. The therapeutic, reciprocating, side-firing, optical fiber is housed in the bottom fixed jaw, while a linear array of four photodiodes and a neutral density filter for diagnostic feedback, is housed in the upper, hinged, active jaw

Figure 5 shows a diagram of a proposed prototype laparoscopic device integrating the transparent quartz optical chamber used in these studies. The upper, movable jaw, pivots on a hinge, and is used to compress the vessel. The incorporation of a transparent quartz chamber may enable the addition of photodiodes (PD), for measuring the optical transmission signal and providing optical diagnostic feedback. The quantity of PDs implemented should encompass the complete fiber scan length to ensure vessel bisection. To protect the photodiodes from excessive laser light, an absorptive neutral density filter may be employed. The bottom, fixed jaw provides therapeutic IR laser delivery, also with a transparent quartz chamber housing the reciprocating, side-firing, optical fiber for vessel sealing/bisection. Future efforts will involve constructing and evaluating this proposed feedback system within a functional laparoscopic quartz jaw prototype.

### Study limitations

One of the limitations in this study was the lack of a fully functional, upper hinged jaw on the device (Fig. 5). A simple transparent optical window was used instead for compressing the tissue, and crudely simulating the optical and thermal properties of a transparent upper jaw. However, both jaws will be transparent in a final design, so this simple approach is still relevant for the optical and thermal studies.

The reciprocating fiber scan length and speed were kept constant in this preliminary study, for simplicity, and to prove feasibility of the simultaneous IR laser sealing and cutting technique. Although the parameters programmed were practical and similar to those used in previous studies [10, 11], a future goal is to customize scan length to match the compressed vessel width. This approach will ensure that excess laser energy is not transmitted around the sample, which is not only inefficient, but may also potentially result in safety issues due to higher device jaw temperatures. More efficient, custom scan lengths may also further lower device temperatures and cooling times, as well as laser treatment times.

It is also important to emphasize that there is significant variation in blood vessel composition (e.g. collagen / elastin ratio) among samples, which factors into the results (e.g. yielding a wide range of burst pressures).

Finally, while the peak temperatures measured in this study for the IR laser vessel sealing device are lower than the peak temperatures reported for conventional electrosurgical and ultrasonic devices, thus producing a better safety profile, there is still the potential for thermal damage to adjacent healthy tissues through accidental contact with the device.

## Conclusion

Simultaneous IR sealing and bisection of blood vessels was conducted using a square quartz optical chamber (2.7 × 2.7 × 25 mm outer dimensions) that can be integrated into a standard 5-mm-OD laparoscopic device. The shortest irradiation time was 5–6 s using 59 W of laser power. Peak temperatures on the external chamber surface measured 103 ^o^C, with a time of 37 s to cool to body temperature. Optical transmission measurements showed a rapid increase and saturation of the photodetector signal upon tissue ablation, which could potentially be used as an optical feedback system to confirm vessel bisection during the procedure. A depiction of the proposed laparoscopic prototype, showcasing the transparent quartz optical chamber employed in this study, was presented. Future work will involve design and construction of a complete prototype device for use during in vivo studies.

## Appendix A: ablation velocity

In ablation models, the postulation of energy equilibrium at the ablation front gives rise to a constant, steady-state ablation velocity [20]. The ablation velocity was determined from the moment of tissue carbonization. Hence, phase B of the transmission signal (Fig. 4), was used to calculate all ablation velocity model variables for blood vessels at a laser wavelength of 1470 nm. Average ablation velocity is approximated by the formula [21]:

$$ ablation velocity v [cm/s] = \frac{f{u}_{a}dkE}{Q}$$

where

$$ E$$ = irradiance [W/cm^2^].

$$ Q$$ = heat of vaporization of water [J/cm^3^] = 2520 J/cm^3^.

$$ {u}_{a}$$ = absorption coefficient of carbonized tissue [cm^−1^] = 21.7 cm^−1^ [21].

$$ d$$ = thickness of carbon layer [cm] = 25 μm [20].

$$ k$$ = augmentation factor from multiple light passes through carbon layer due to light scattering and total internal reflection

$$ f$$ = apparent efficiency of converting absorbed energy into ablation = 0.45 [22].

In the transmission studies, incident laser power was 6.5 W with a circular beam diameter of 0.560 mm. Irradiance (E) is calculated as:

$$\eqalign{& {\rm{E}}\; = \;{{6.5} \over {\left( {3.14} \right)\left( {0.28} \right)2}}\; = \;26.4\;{\rm{W}}/{\rm{m}}{{\rm{m}}^2}  \cr &  = \;2640\,\;{\rm{W/c}}{{\rm{m}}^2} \cr} $$

$$ v$$ was calculated using total time of phase B. Average time of phase B (*n* = 8) transmission signal was 0.9 s. Consequently, for tissue compressed to 0.4 mm:

$$v = {{0.4\,\,\text{mm}} \over {0.9\,\,\text{s}}} = \;0.44\;{\rm{mm/s}}\;{\rm{ = }}\;{\rm{0}}{\rm{.044}}\;{\rm{cm/s}}$$

The missing variable in this model for ablation velocity of the vessel at 1470 nm is the augmentation factor, $$ k$$, that was calculated using the velocity equation as:

$$ k=\frac{vQ}{f{u}_{a}dE}= \frac{\left(0.044\right)\left(2520\right)}{\left(0.45\right)\left(21.7\right)\left(0.0025\right)\left(2640\right)}=1.7$$
